# P-681. Evaluation of Clinical Characteristics and Antimicrobial Stewardship Interventions in Mycoplasma pneumoniae Infections at a Community Teaching Hospital

**DOI:** 10.1093/ofid/ofaf695.894

**Published:** 2026-01-11

**Authors:** Paula A Politis, Thomas M File, Michael Tan, Matthew England

**Affiliations:** Summa Health System, Akron, OH; Summa Health , Akron, OH; Summa Health System, Akron, OH; Summa Health, Akron, Ohio

## Abstract

**Background:**

Patients admitted with community-acquired pneumonia (CAP) are empirically treated with broad-spectrum antibiotics due to challenges identifying causative organisms. *Mycoplasma pneumoniae* is a common atypical pathogen but has historically been difficult to detect. Implementation of rapid molecular diagnostics at our institution improved detection of *M. pneumoniae*, supporting more targeted interventions by the Antimicrobial Stewardship Program (ASP). A recent rise in *M. pneumoniae* cases presented an opportunity to assess clinical characteristics, management, and stewardship impact.

**Figure:**
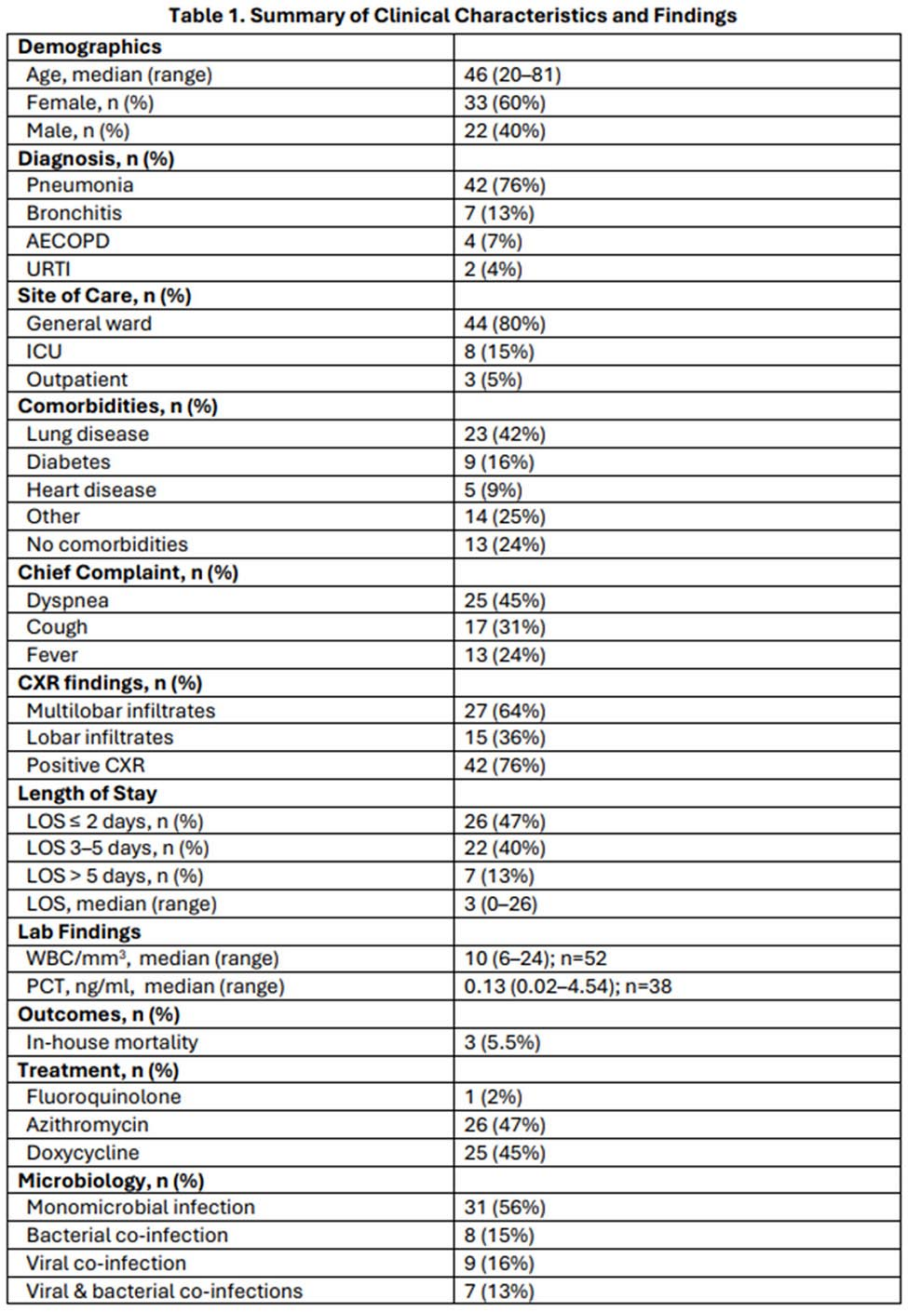

**Figure:**
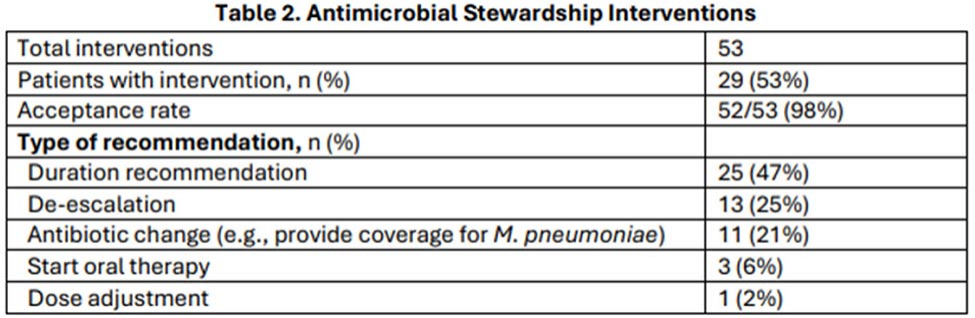

**Methods:**

We performed a retrospective review of patients admitted to our institution from June 2024 – March 2025 with *M. pneumoniae* identified by PCR, including the Respiratory Pathogen Panel (RPP) and the Pneumonia Panel (PN). Data collected included demographics, comorbidities, imaging, co-infections, antimicrobial therapy, ASP interventions, and outcomes.

**Figure:**
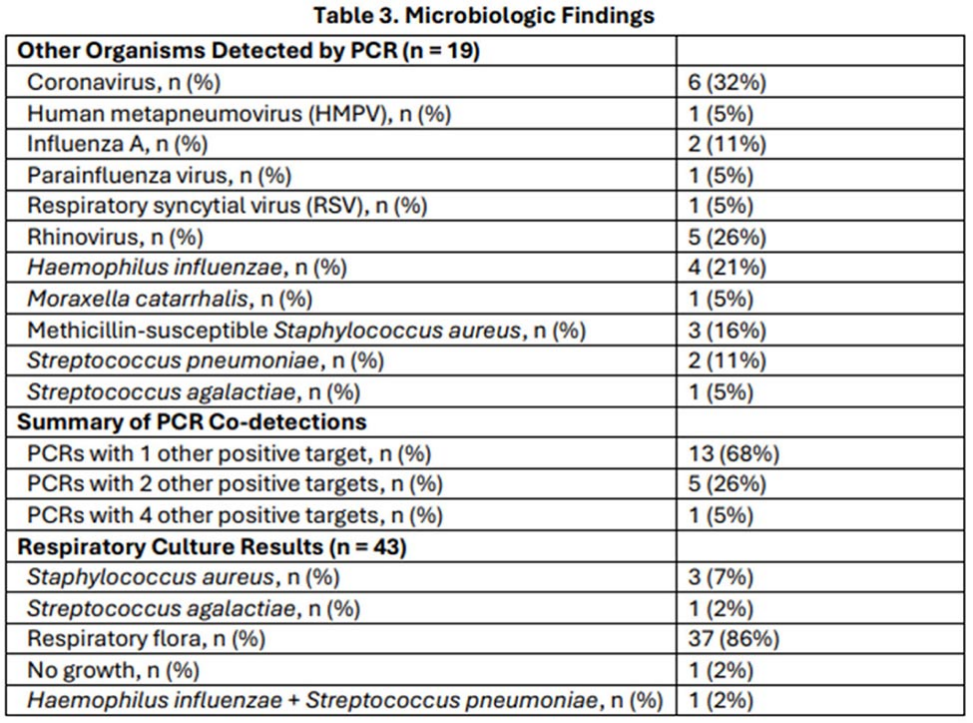

**Results:**

55 patients were reviewed (median age 46 years, 60% female), with most diagnoses occurring in fall months (September–November). Dyspnea and cough were the most common complaints. Pneumonia was diagnosed in 76% of cases and 19% of those were admitted to the ICU. Potential co-pathogens were observed in 44%, including bacterial (14%), viral (16%), and mixed (13%) cases. Median length of stay was 3 days; mortality was 5.5%. Most patients received azithromycin (47%) or doxycycline (45%). The ASP provided 53 interventions across 29 patients, including 7 in which atypical coverage was initiated. Median procalcitonin and WBC were 0.13 and 10, respectively. Additional results in Tables 1–3.

**Conclusion:**

A marked rise in *M. pneumoniae* cases was observed over the review timeframe, consistent with known cyclical trends and potentially influenced by reduced public health mitigation measures following the COVID-19 pandemic. Most infections occurred in younger female patients with typical clinical findings and were managed on general medical floors with short stays. Rapid diagnostic testing facilitated early identification and enabled ASP-led optimization of antimicrobial use. These observations highlight the importance of ongoing surveillance of atypical pathogens and the role of stewardship in improving targeted therapy.

**Disclosures:**

Thomas M. File, Jr., MD, MSc, MACP, FIDSA, Merck: Advisor/Consultant|MicroGenDx: Advisor/Consultant|Paratek: Advisor/Consultant|Shionogi: Advisor/Consultant|Shionogi: Honoraria|ThermoFisher: Advisor/Consultant|ThermoFisher: Honoraria

